# Human land‐use effects on mammalian mesopredator occupancy of a northeastern Connecticut landscape

**DOI:** 10.1002/ece3.9015

**Published:** 2022-07-03

**Authors:** Kimberly M. Zamuda, Marlyse C. Duguid, Oswald J. Schmitz

**Affiliations:** ^1^ School of the Environment Yale University New Haven Connecticut USA

**Keywords:** camera trapping, carnivore conservation, forest management, land use, timber harvesting

## Abstract

Mammalian mesopredators—mid‐sized carnivores—are ecologically, economically, and socially important. With their adaptability to a variety of habitats and diets, loss of apex predators, and forest regrowth, many of these species are increasing in number throughout the northeastern United States. However, currently the region is seeing extensive landscape alterations, with an increase in residential and industrial development, especially at the expense of existing forest and small‐scale farmland. We sought to understand how important an existing mosaic of working lands (timberland and farmland) in a forested landscape is to mesopredator species. We did this by studying mesopredator occupancy across three land uses (or habitat types): forest reserve (protected), timber harvest (shelterwood cuts), and field (both crop yielding and fallow) in and around a 3200‐ha forest in northeastern Connecticut. We examined coyote (*Canis latrans*), bobcat (*Lynx rufus*), fisher (*Pekania pennanti*), and raccoon (*Procyon lotor*) occupancy using paired camera traps across juxtaposed reserve, shelterwood, and field units from April 2018 to March 2019. We created a priori habitat variable models for each species and season, as well as analyzed the impact of habitat types on each species. Throughout the year bobcats were positively associated with foliage height diversity and had the highest use in shelterwoods and lowest use in fields. Land use utilization varied seasonally for coyotes and raccoons, with higher use of fields than reserves and shelterwoods for half the year and no difference between land uses and the other half. Both species were not strongly associated with any particular habitat variables. Reserve forest was moderate to highly used by all species for at least half the year, and highly use year‐round by fishers. Our findings reveal that a mosaic of intact forest and working lands, timber harvest, and agriculture can support mesopredator diversity.

## Graphical abstract

The northeastern United States is currently experiencing extensive landscape alterations at the expense of existing forest and small‐scale farmland. We sought to understand the importance of the existing mosaic of working lands (timberland and farmland) in a forested landscape to mesopredator species. Our analysis, evaluating yearlong mesopredator occurrences among different land uses, reveals this mosaic of forest and working lands supports and plays a role in mesopredator diversity.

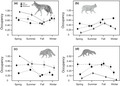

## 1 INTRODUCTION

The landscape in the northeastern USA has undergone numerous land‐use changes over the course of the past 300 years. Particularly dramatic has been the landscape‐scale clearing of forest ecosystems for colonial agriculture, followed by forest recovery after agricultural abandonment (Foster & Aber, 2004; Foster, 1992). This dynamic has caused an associated loss and recovery of numerous wildlife species, notably large and mid‐sized mammalian carnivores (Farrell et al., 2018; Foster et al., 2002; Litvaitis et al., 2006; Ray, 2010), which collectively are considered to be vulnerable to habitat alteration, fragmentation, and loss (Carrasco et al., 2009; Farrell et al., 2018; Long et al., 2011; Ripple et al., 2009). Forest ecosystem recovery has now resulted in the recovery of many mesopredator species—mid‐sized (1–15 kg) carnivores (Prugh et al., 2009; Roemer et al., 2009)—that are native to the region, for example, bobcats (*Lynx rufus)*, foxes (*Vulpes vuples and Urocyon cinereoargenteus*), raccoons (*P. lotor*), and fisher (*Pekania pennanti)* (Ray, 2010). As well, it has facilitated the range expansion of other native mesopredators, for example, coyotes (*Canis latrans)* (Foster et al., 2002; Kays et al., 2008; Ray, 2010). The ability of mesopredators to thrive in this landscape may be further abetted by release from predation by large apex predators that have been and continue to be extirpated (Ray, 2010; LaPoint et al., 2015), a phenomenon known as mesopredator release (Richie and Johnson, 2009; Prugh et al., 2009).

Mesopredators occupy a wide range of habitats and have varied diets (Buskirk, 1999; Roemer, 2009). They thereby can have ecologically diverse functional roles, including regulation of prey abundances, which can have cascading effects on vegetation abundance and ecosystem nutrient cycling, on zoonotic disease spread (Prugh et al., 2009; Roemer et al., 2009), as well as potentially creating human–wildlife conflict (Prugh et al., 2009). These effects may, however, be mediated by bottom‐up factors such as habitat availability, ecosystem productivity, and human‐built features (e.g., roads) that determine mesopredator occurrence across landscapes (Crimmins et al., 2016; Farrell et al., 2018; O'Connor & Rittenhouse, 2017; Roemer et al., 2009).

This may be especially relevant in northeastern rural landscapes which are a complex mosaic of working lands (i.e., timber and farmland) embedded in predominantly second‐growth forested landscapes. Agricultural and timber lands have important roles in local economics, food security, and climate change mitigation (Foster, 2017; Lopez et al., 2017). Over several decades Connecticut has lost significant forest and agricultural land to development and suburbanization (Arnold et al., 2020). Wildlife conservation efforts in the region are focused on preserving mature, intact forests, with less emphasis paid to timber and agricultural lands. With this, wildlife research is focused on the impacts of fragmentation due to roads, industrial, and residential development on wildlife communities in forests (Forman et al., 2003; Kluza et al., 2000). However, much less is known about the role of working lands in the landscape or the impacts of their loss. Overall, these land‐use changes may alter the habitat for mesopredators and potentially shape their occurrence and persistence on the landscape (Farrell et al., 2018).

Conservation planning is often informed by occupancy analyses, which have been used to assess mesopredator–habitat associations, use of an area, and predict future species distributions as the availability of habitat types' changes (Fuller et al., 2016; Moreira‐Arce et al., 2016; O'Connor & Rittenhouse, 2017; Linden et al., 2017; Litvaitis et al., 2006; Long et al., 2011; Reed et al., 2017). But measuring occupancy and use merely with coarser scale descriptors such as habitat type will be insufficient for understanding the basis for variation in mesopredator–habitat associations across landscapes. This is because the probability of occupancy and use within any habitat type may vary with fine‐scale environmental variables including how the nature of land‐use influences vegetation structure and cover within a habitat type, adjacency of habitat type to other land use, the distance of habitat type to waterbodies, and human‐built infrastructure (Crimmins et al., 2016; Gompper et al., 2016; Gese & Thompson, 2014; Moreira‐Arce et al., 2016; Reed et al., 2017). Additionally, single‐season studies, frequently used in wildlife research, are often insufficient to accurately inform conservation plans. Full‐year or multiple season studies are required to account for seasonal variation in occupancy and the use of fine‐ and coarse‐scale variables, and habitat types (Ikeda et al., 2016; Lesmeister et al., 2015; Ray, 2010; Zielinski et al., 2015).

We report on mesopredator occupancy analysis for coyote (*Canis latrans*), bobcat (*Lynx rufus*), fisher (*Pekania pennanti*), and raccoon (*Procyon lotor*) in a mosaic of protected forest interspersed with working forest land for timber production and smallholder agriculture in northeastern Connecticut, USA. Our goal was to evaluate how working lands in an otherwise forested landscape influenced mesopredator occurrence over the course of a year. We leveraged an ongoing mosaic of land uses as an observational experiment. The study area provides the ability to evaluate and compare how timber management and smallholder agriculture influence mesocarnivore occupancy relative to protected (reserve) forest stands. We further utilized occupancy analysis to ascertain whether habitat variables can lead to a predictive understanding of variation in mesopredator–habitat associations across the managed landscape (Gorosito et al., 2018). Our selection of habitat variables was species‐dependent and motivated by natural history insights about habitat variables with which these mesopredators are associated. Accordingly, we hypothesized that coyotes would be positively associated with distance to skid roads, public roads, percent forest (based on increased ease of movement and protected denning locations), and negatively associated with canopy cover, due to lower prey availability (Atwood et al., 2004; Hinton et al., 2015; Kays et al., 2008; Lesmeister et al., 2015). We hypothesized that bobcats would be negatively associated with distance to road and canopy cover (based on preference for unfragmented habitat and lower prey availability), and positively associated with foliage height diversity and distance to nearest stream/river, due to more food resources (Broman, 2014; Donovan et al., 2011; Litvaitis, 2001, Litvaitis et al., 2006). We hypothesized that fishers would be positively associated with canopy cover, foliage height diversity, and snag density, based on prey availability and den site requirements (Buskirk & Powell, 1994; Degraaf & Yamasaki, 2001; Gibilisco, 1994; Krohn et al., 1995; Ray, 2000; Zielinski et al., 2010). We hypothesized that raccoons would be positively associated with percent wetland (based on increased food resources), and negatively associated with percent forest and distance to road, due to reduced food resources and barriers to movement (Beasley et al., 2011; Chamberlain et al., 2003; Owen et al., 2015; Pedlar et al., 1997; Whitaker & Hamilton, 1998).

The study provides quantitative scientific insight to support landscape management planning to balance human land uses and mesopredator habitat conservation in a New England Landscape. Specifically, we reveal (i) how forest harvesting and management, and smallholder farmland impact mesopredators and (ii) seasonally, which habitat variables are most important for mesopredator species conservation.

## 2 MATERIALS AND METHODS

### 2.1 Study area

The study was conducted in and around the Yale Myers Forest (YMF), a 3200‐ha research and demonstration forest in northeastern Connecticut (418570 N, 728070 W). This forested landscape is covered by second‐growth central hardwood‐hemlock pine, which resulted from old‐field pine succession after the abandonment of agricultural lands in the 1800s (Foster, 1992; Meyer & Plusnin, 1945). The forest is composed of many dominant tree species, including eastern white pine (*Pinus strobus*) eastern hemlock (*Tsuga canadensis*), red oak (*Quercus rubra*), multiple species of maple (*Acer* spp.), birch (*Betula* spp.), and hickory (*Carya* spp.) and more than 200 species of understory plants (Duguid et al., 2016; Meyer & Plusnin, 1945). The current land use includes active management for both timber and research. For management, the forest is designated into 8 divisions of about equal size (on average around 400 ha, ranging from 310 to 485 ha). The landscape rises 170–300 m above sea level, with a ridge and valley topography. The temperature ranges from 27°C during summer to −4°C during winter (NOAA, 2019). In the following, the terms land‐use type and habitat type are synonymous. We use land‐use type to refer to human occurrence in and use of different parts of the landscape; and habitat type to refer to (non‐human) mesopredator species occurrence and use of the parts of the landscape.

YMF and the surrounding landscape is a mosaic of land‐use (aka habitat) types including protected forest reserves (including state parks and forests), managed forest, small landholder agricultural and abandoned fields, and fragmented residential lands. Our study occurred within YMF and on adjacent private and public land. The private land‐use consisted of small (6.07–20.2 ha) agricultural, primarily corn, and non‐agricultural fields (fields). One‐third of the YMF land base is an unharvested protected forest reserve (with areas varying from 10.37 to 18.3 ha) and the remaining two‐thirds is managed for timber production (with shelterwood stands varying from 5.45 to 16.9 ha). Forest reserves are mature, mostly second‐growth hardwood forest stand, generally, between 100 and 120 years old. While some thinning may have been done historically, none had been harvested by humans for at least 30 years.

Within the managed forest areas irregular shelterwood silvicultural systems are the primary regeneration strategy and have been used in YMF since the 1990s. Shelterwood harvests promote regeneration by harvesting 50–80% of the basal area leaving large, evenly spaced legacy trees that range in number, basal area, species, and diameter, varying with site and prescription (Ashton & Kelty, 2018). Since these shelterwood regeneration harvests alter forest structure by removing the majority of canopy trees, these stands are initially converted into the early successional habitat but are dynamic systems. In this study, shelterwoods varied between 2 and 15 years since they were harvested.

### 2.2 Study design

**FIGURE 1:**
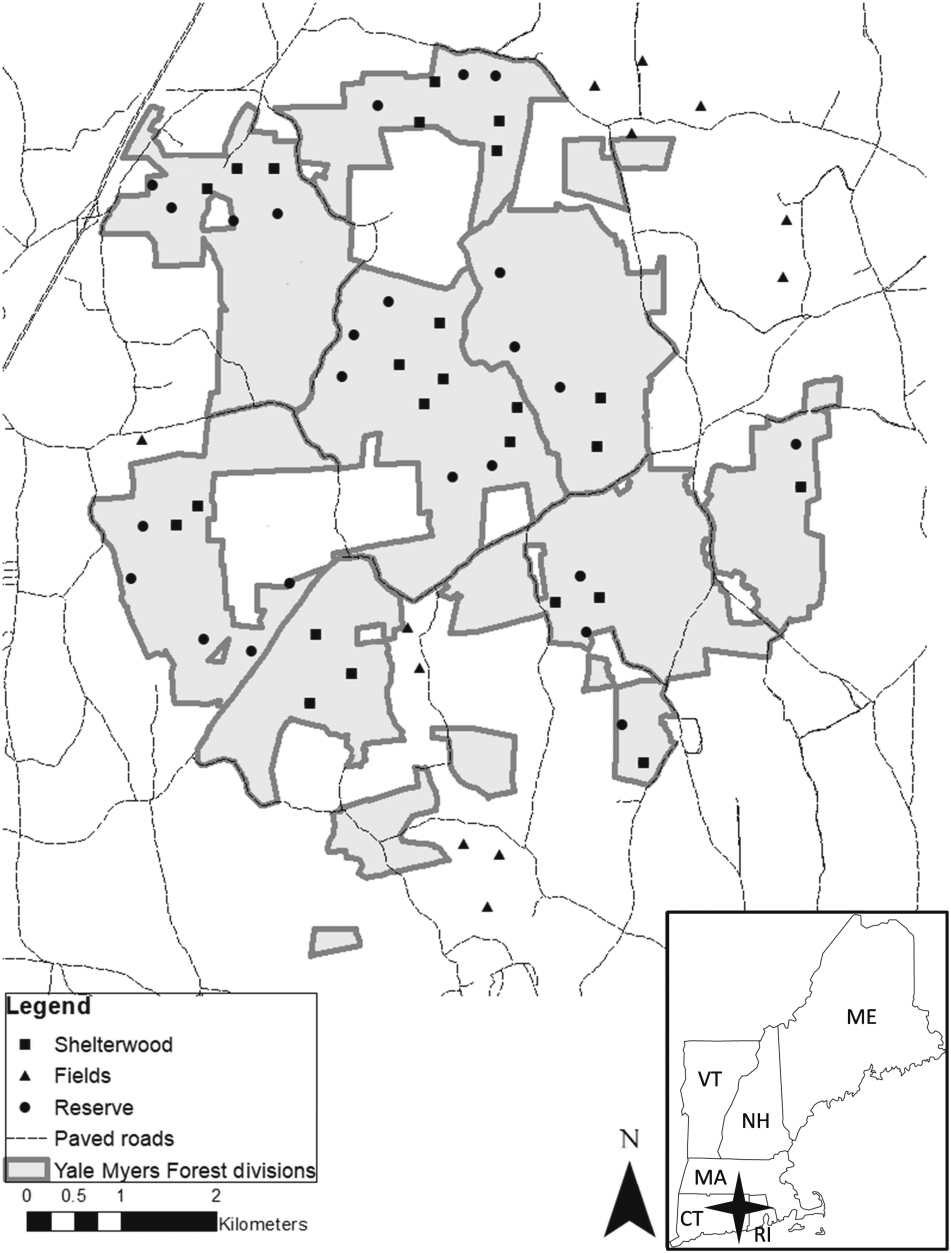
The camera trap sites in and around Yale Myers Forest, northeastern Connecticut. Twenty‐four sites were in shelterwoods, twenty‐four were in reserve forest, and twelve were in fields

We evaluated mesopredator occupancy among the three general human land‐use types: agricultural fields, forest reserves, and shelterwood harvests, each occurring as an individual, replicate units within several forest management divisions across the YMF landscape. We selected 24 camera trapping sites from available forest reserve units, 24 camera trapping sites from available shelterwood units, and used 12 camera trapping sites from all available agricultural field units. In our study, reserve reflects mature forest conditions arising from over a century of forest recovery, shelterwood reflects forest management‐induced early successional forest, and field reflects centuries of reversal in forest habitat availability through land conversion for alternative uses. We evaluated occupancy among land‐use units arrayed to ensure spatial adjacency of at least two different land‐use types among replicate camera trapping sites (Figure 1). Our adjacency criterion resulted in the use of shelterwood harvests that were proximal to forest reserves, and fields that were adjacent to reserves. Each adjacent grouping of camera trapping sites was on average 1000 m apart from other replicate groupings of sites and at least 250 m from the nearest public paved road (Figure 1.)

### 2.3 Mammal survey data

We sampled mesopredators using intensive camera trapping. We sampled for an entire year from the beginning of April 2018 until the end of February 2019. We divided the year into four time periods to reflect different seasonal conditions determining mesopredator activity cycles in southern New England (O'Connor & Rittenhouse, 2017): spring (April–May), summer (June–August), fall (September–November) and winter (December–February). Within each season, we sampled on three replicate sets of 21 survey days. We sampled new adjacent land‐use units, selected from across the landscape, in each 21‐day period to maximize the number of sites surveyed. This led to 24 camera trapping sites sampled during each 21‐day period (Franklin et al., 2019; Lesmeister et al., 2015). We used 50 paired digital passive infrared motion‐sensing cameras with infrared flash (Bushnell Trophy Cam HD, Bushnell, Kansas) to sample animal presence and estimate occupancy.

We positioned the cameras to detect a variety of mesopredator species including coyote, bobcat, fisher, raccoon, red fox (*V. vulpes*), gray fox (*U. cinereoargenteus*), and skunk (*Mephitis mephitis*) following established camera‐trapping protocols used to detect these species in other study locations (Kelly & Holub, 2008; Lesmeister et al., 2015; Gompper et al., 2016; O'Connor & Rittenhouse, 2017). Two cameras were placed together at each site (within 25 m of each other) to increase detection likelihood (O'Connor et al., 2017; Pease et al., 2016). The cameras were tied to trees between 0.4 and 0.6 m above the ground, facing away from the sun. Vegetation and sticks were cleared from the camera view to reduce the number of false triggers caused by swaying in wind. We checked the cameras and moved them to new sites three times every season, resulting in 3, 21‐day sampling periods within each season (see S2 for diagram). Daily photographic evidence of species presence was recorded for each site. For analysis, the 21 survey days were condensed down to 7 survey days, with every 3 days grouped together into a single survey, consistent with other protocols (Cove et al., 2017). For illustration, a presence (1)/absence (0) sequence of 100‐000‐001 (9 surveys) was reduced to 1–0‐1 (3 surveys). We used these data to estimate the detection probability and occupancy at a site for each species for each season (MacKenzie et al., 2006).

Overall, we sampled a total of 15,183 camera trap nights between April 2018 and February 2019, (where a camera trap night is defined as a 24 h period of data collection from the cameras). This resulted in 462 photographic captures for coyotes, 422 for raccoons, 176 for bobcats, and 56 for fishers. We excluded other potential mesopredator species, including gray fox, red fox, striped skunk, and otter from further analyses because the low numbers of photographic captures for these species precluded calculating reliable occupancy estimates for them.

### 2.4 Habitat variables

We measured fine‐scale habitat characteristics of land‐use types and averaged for every season. At each camera trap location, we measured canopy cover, foliage height diversity, the volume of coarse woody debris (CWD), and snag density. We measured canopy cover with a convex spherical densitometer at eight locations in each camera trap site (one at each of the two cameras, and three in different random cardinal directions 100 m from each camera). We measured foliage height diversity using a 2.5 m tall vertical pole (partitioned with a bright ribbon every 0.5 m). For each site, at eight locations we held a pole 100 m away from each camera in random cardinal directions and recorded the percent vegetation in each size class (0–1 m, 1–2 m, 2–2.5 m, 2.5 m+). We quantified foliage height diversity using the Shannon index (−∑ *p*_*i*_ ln (*p*_*i*_) where *p*_*i*_ is the average percent vegetation of the 8 measurements at each height, summed over the 4 height categories (Nudds, 1977). We sampled CWD using the line‐intercept method. We sampled along with four, 100 m transects emanating from the camera trap site center using random bearings and measuring all CWD (>10 cm in diameter and >1 m in length) for length and diameter at intersect of the transect. The volume of CWD was calculated using the formula V = π^2^ ∑(*d* ^2^ / 8 L), where *d* is the diameter of wood at the intersection and *L* is the length of the transect (McCurdy & Stewart, 2005). We recorded the number of snags (dbh greater than or equal to 8 cm and at least 2 m in height) within a 50 m radius circle plot of the camera trap site center and within three 50 m radius plots 100 m away from the center in random cardinal directions. From US data, we recorded precipitation and temperature for each trap day (US Climate Data 2019).

We quantified 15 additional local and landscape (coarse‐scale) variables in 1 km circular buffers surrounding each camera trapping site in R Studio (R 3.5.1 [ R Core Development Team, 2020]) using the packages rgdal, FedData, raster, maptools, rgeos, spData, and gdistance (Bivand and Levin‐Koh, 2019; Bivand, Keitt, & Rowlingson, 2019; Bivand, Nowosad, & Lovelace, 2019; Bivand and Rundel, 2019; Bocinksky et al., 2019; Etten, 2018; Hijmans, 2019). We characterized land cover using eight variables. These were percent forest cover, percent conifer forest, percent deciduous forest, and percent mixed forest; percent developed land, percent agricultural/field, percent wetland, and distance to nearest stream or river. Each of these variables were extracted individually from the National Land Cover Database (NLCD, 2011). Elevation data for each site were also extracted from the 1 km buffers using data from the National Elevation Database (NED, 2018). Anthropogenic features included distance to nearest public road and distance to nearest forest harvesting skid road from each site (road data were obtained from the YMF database). The percent crop type in agricultural fields was also extracted from each 1 km buffer from the United States Department of Agriculture Cropland Data Layer.

### 2.5 Statistical analyses

#### 2.5.1 Detection probability and species associated habitat variable analysis

We used single‐species, single‐season models in the program PRESENCE to estimate mesopredator detection probabilities (*p*) and occupancy (*psi*) (s). We applied a stepwise process consistent with approaches in previous studies (Cove et al., 2012; Franklin et al., 2000; MacDougal et al., 2022; Lesmeister et al., 2015; Long et al., 2007; Long et al., 2011; Lombardi et al., 2017). For each species, we modeled detection probability and then used the top detection variables to derive occupancy estimates. We estimated the detection probability for each species to adjust occupancy for imperfect detection of each species. We treated environmental conditions that could influence species' activity and hence detection (daily temperature, precipitation, and previous detection at a sampling site) as covariates to predict the detection probability (p) of a species (Gompper et al., 2016; Fuller et al., 2016; Pease et al,. 2016). We also included fine‐scale habitat variables that could impact species' detection: foliage height diversity and canopy cover (Eng & Jackson 2019; Magle et al., 2016). Daily temperature and precipitation data were taken from US Climate data ((US Climate Data 2019). All continuous variables used to estimate detection probability and occupancy were standardized as z‐scores.

D For detection analysis, we held occupancy constant for all models, assuming that occupancy did not vary across sites due to environmental variables (Lesmeister et al., 2015). In the null model, we held occupancy and detection probability constant. We evaluated models based on Akaike's Information Criterion (AIC) values and model weight (MacKenzie et al., 2006). We hypothesized that precipitation and temperature would negatively relate to all species detection; we hypothesized that previous detection would positively relate to coyotes and raccoons, and negatively related to bobcats and fishers. These covariates have been previously shown to affect mesopredator species detection (Duscher et al., 2017; Gompper et al., 2016; Lesmeister et al., 2015; Pease et al., 2016; Madsen et al., 2020; Melville et al., 2020; Shivik et al., 1997). We used the best (lowest AIC) species‐specific p models to determine which habitat variables and habitat type (land use) influenced species occupancy (interpreted as use of an area) (Burnham and Anderson 2002).

We individually evaluated how different habitat variables and habitat types influenced species occupancy estimates (psi) (Mackenzie et al., 2002, 2006). Multi‐model inference via model selection is a common approach to determining habitat variable associations with species occupancy (Stephens et al., 2005; Aho et al., 2014; Bailey et al., 2014). For habitat variables analysis, we built habitat models using a priori fine‐ and coarse‐scale habitat variables (See S3), motivated by a priori hypotheses about habitat variables that are known (from previous studies) to impact mesopredator occupancy, in an effort to root our study in biology (Whittingham et al., 2006). For further evaluation of habitat variable importance, we considered variables significant when the 95% confidence interval (CI) did not cross zero (Cove et al., 2019; Eng & Jachowski, 2019; Lesmeister et al., 2015; Long et al., 2011; Macdougall & Sanders, 2022; Wilson & Schmidt, 2015). Next, to evaluate whether habitat type (reserve, shelterwood, and field) impacts mesopredator occupancy estimates, we ranked three models for each species and season. The models were (1) habitat type; (2) habitat type and the variables included in the top occupancy model for each season and species (determined by the habitat variables analysis); and (3) the variables included in the top occupancy model from habitat variables analysis). For this analysis, when habitat type occurred in the top model for a species, we used odds ratios (e^β) and odds ratio 95% CI to assess the significance between habitat types. We considered it significant when the 95% CIs did not cross one (Ceradini et al., 2021; Lombardi et al., 2020). For both analyses, we ranked models based on AIC values and model weights; we considered models within two AIC units of the top model (Burnham & Anderson, 2002). Given species home ranges and our study site size, we interpreted occupancy (*psi*) estimates as estimates of species use of an area in our results and discussion.

We ran goodness‐of‐fit tests on the global models, using 10,000 parametric bootstraps, to determine if there was evidence of overdispersion (Mackenzie & Bailey, 2004). We also confirmed that multicollinearity was not an issue by testing Variance Inflation Factors (VIF) (all variables in our models had VIF values <3) (Zuur et al., 2009).

## 3 RESULTS

### 3.1 Mesopredator detection and associated habitat variables

Estimates of detection probability (±SE) varied between species, but tended to be consistent across seasons within each species (Table 1 and see supplemental material: S7). The occupancy models showed no evidence of overdispersion (ĉ<1).

Within seasons bobcat use of an area was associated with several habitat variables (foliage height diversity, distance to public road, and canopy cover) (Table 3). Bobcats were positively associated with foliage height diversity during spring, summer, fall, and winter (Table 3). For both spring and fall seasons, our top model contained only foliage height diversity (Table 2). Foliage height diversity and distance to public roads comprised the top model for the summer season, where bobcats were negatively associated with public roads (Table 2; Table 3). During the winter season, public roads were in the third‐ranked model (with foliage height diversity). However, the effect was weak and there was not a significant relationship with occupancy, as the 95% CI overlapped with 0 (S4). Bobcats were also negatively associated with canopy cover during the winter—comprising the second‐ranked model with foliage height diversity (Table 2). Bobcats are also potentially associated with canopy cover in the summer (the 95% CI slightly overlapped with 0) (Table 3).

**TABLE 1:** Estimate of mesopredator detection probability (±SE)

Spring	Season	Detection probability (± SE)
Coyote	Spring	0.39 ± 0.02
	Summer	0.30 ± 0.03
	Fall	0.37 ± 0.064
	Winter	0.35 ± 0.03
Bobcat	Spring	0.25 ± 0.02
	Summer	0.27 ± 0.07
	Fall	0.23 ± 0.08
	Winter	0.21 ± 0.033
Raccoon	Spring	0.30 ± .032
	Summer	0.33 ± 0.02
	Fall	0.22 ± 0.063
	Winter	0.25 ± 0.04
Fisher	Spring	0.22 ± 0.092
	Summer	0.25 ± 0.042
	Fall	0.23 ± 0.06
	Winter	0.24 ± 0.03

**TABLE 2:** Ranked most supported occupancy models (within 2 AIC units of the top model) for mesopredator species in and around Yale Myers Forest, Connecticut, USA. The detection covariates included in each model were from the best (most parsimonious) detection probability model determined by model selection for each species. For the full model results see supplemental materials (S3)

Season	Model^a^	AIC^b^	ΔAIC	*w* ^c^	K^d^
Bobcat					
Spring	FHD	559.20	0	0.487	4
Summer	FHD + DPR	510.2	0	0.384	5
	FHD	510.36	0.16	0.331	4
	FHD + DPR + CC	511.74	1.54	0.183	6
Fall	FHD	523.1	0	0.53	4
Winter	FHD	499.6	0	0.335	4
	FHD + CC	500.38	0.78	0.319	5
	DPR + FHD	501.57	1.97	0.128	5
Coyote					
Spring	CC	724.87	0	0.467	3
	CC + DPR	726.74	1.87	0.255	4
Summer	DSR + CC	756.33	0	0.432	5
	CC	756.78	0.45	0.404	4
Fall	FOR + CC + DSR	710.24	0	0.254	6
	CC	711.18	0.94	0.197	4
	(.)	711.56	1.32	0.188	3
Winter	(.)	742.36	0	0.249	3
Fisher					
Spring	CC + FHD	325.94	0	0.301	8
	CC	326.15	0.21	0.256	7
	FHD	326.51	0.57	0.224	7
Summer	SD	305.88	0	0.374	4
	SD + FHD	306.33	0.45	0.328	5
Fall	SD	312.34	0	0.523	4
	CC+ SD	313.98	1.64	0.287	5
Winter	FHD + SD	348.23	0	0.287	6
	FHD	348.61	0.38	0.259	5
Raccoon					
Spring	WET	274.92	0	0.243	4
	DPR + WET	276.12	1.2	0.181	5
Summer	DPR	268.33	0	0.278	4
	DPR + WET + FOR	269.36	1.03	0.216	6
	DPR + WET	269.9	1.57	0.171	5
Fall	FOR	250.24	0	0.192	4
	FOR +WET	252.07	1.83	0.157	5
Winter	DPR + WET	285.75	0	0.210	5
	(.)	286.01	0.26	0.196	3
	DPR + WET + FOR	286.72	0.97	0.118	6

^a^ CC, canopy cover; DPR, distance to a public road; DSR, distance to skid road; FHD, foliage height diversity; FOR, percent forest; SD, snag density; WET, percent wetland.

^b^ Difference in Akaike's Information Criterion from the top model to the current model.

^c^ Model weight (model probability).

^d^ Number of model parameters.

^e^ −2Log(Likelihood), measure of model fit.

**TABLE 3:** Estimated coefficients for habitat variables associated with mesopredator species by season from our top‐ranked habitat variable models. Only significant variables (based on a 95% confidence interval that excludes zero) are shown with their associated unstandardized coefficients and standard errors. For full model results see supplemental materials (S4)

Season	Species	Habitat variable	Estimated coefficients (SE)	95% confidence interval
Spring	Bobcat	Foliage height diversity	0.352 (0.102)	0.152–0.552
	Coyote	Canopy cover	0.744 (0.305)	0.146–1.342
	Raccoon	Percent wetland	0.473 (0.208)	0.065–0.881
	Fisher	Canopy cover	0.635 (0.295)	0.056–1.213
		Foliage height diversity	−0.531 (0.259)	−1.038 – −0.022
Summer	Bobcat	Foliage height diversity	0.923 (0.185)	0.559–1.286
		Distance to public road	0.924 (0.401)	0.139–1.710
	Coyote	Canopy cover	0.955 (0.391)	0.190–1.721
	Raccoon	Distance to public road	−0.24 (0.102)	−0.439 – −0.040
	Fisher	Foliage height diversity	2.46 (0.2)	0.108–4.812
		Snag density	0.654 (0.328)	0.010–1.297
Fall	Bobcat	Foliage height diversity	0.623 (0.185)	0.259–0.987
	Raccoon	Percent forest	−1.169 (0.441)	−2.03 – −0.303
	Fisher	Snag density	0.928 (0.45)	0.046–1.810
Winter	Bobcat	Canopy cover	−0.507 (0.203)	−0.904 – −0.109
		Foliage height diversity	0.739 (0.356)	0.041–1.436
	Fisher	Foliage height diversity	0.493 (0.2)	0.101–0.884

Habitat variables associated with fisher use of an area were foliage height diversity, snag density, and canopy cover. During the summer and fall, fishers were positively associated with snag density, with the top‐ranked model for both these seasons consisting of only snag density (Table 2). Our top model for winter also included snag density, though the effect of this variable was weak as the 95% CI overlapped with 0 (S4). Fisher use was positively associated with foliage height diversity during the spring, summer, and winter, and with canopy cover in the spring (Table 3). The top fisher model for spring contained foliage height diversity and canopy cover (Table 2).

Coyote use of an area was positively associated with canopy cover in both the spring and summer (Table 3). For the spring season, our top coyote model contained only a canopy cover (Table 2). Canopy cover and distance to public road comprised our second‐ranked model for spring, however, only canopy cover had a strong relationship with coyote occupancy (Table 3). In the summer, canopy cover and distance to skid roads made up our top model, with only canopy cover as the second‐ranked model (Table 2; S3). Coyotes were potentially positively associated with skid roads in the summer, but perhaps weakly so given the slight overlap of the confidence interval with zero (S4). The null model was the top‐ranked coyote model for the winter season, and the third‐ranked model during the fall. During these two seasons, coyotes were not associated with any habitat variables, as all 95% CIs overlapped with zero (S4).

Raccoon use of an area was seasonally associated with different habitat variables. During spring raccoons were positively associated with percent wetland, and our top model for this season solely contained wetlands (Table 2, Table 3). The top model for winter also included wetlands, as well as the second‐ranked models for both summer and fall, though the strength of this relationship is weak (S3; S4). During the fall, the most important predicator was the percent forest; raccoons were negatively associated with this variable (Table 2). Distance to public roads alone made up our top model for summer, as well as the second‐ and third‐ranked models. Raccoon use was positively associated with roads in the summer and potentially during the spring, as the 95% CIs on coefficient only marginally overlapped zero (S4). For the winter, the null model had similar support as the top‐ranked model (wetlands, forest, and distance to public roads), and raccoons did not show significant association with any habitat variables (S3; S4).

### 3.2 Mesopredator habitat type associations

**FIGURE 2:**
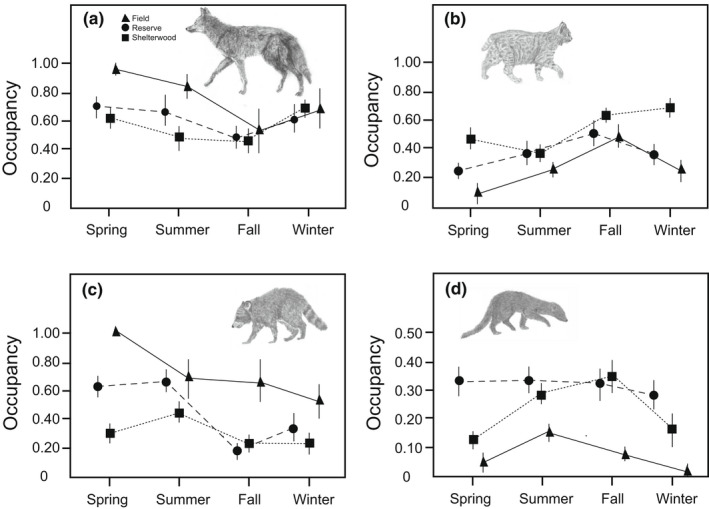
Model averaged occupancy estimates, interpreted as the probability of species use of an area, across seasons for (a) coyote, (b) bobcat, (c) raccoon, and (d) fisher mesopredators in three different habitat types in a managed and developed mixed‐hardwood forest landscape in northeastern Connecticut. Mesopredator species occupancy estimates represent probability (0–100%). Values are mean ± one standard error

Model averaged occupancy estimates varied for species across different habitats and seasons (Figure 2). Habitat type (reserve, field, and shelterwood) had a differential influence on species use of an area, with more impact on certain species than others (S5). Bobcats had the highest average use estimates during the fall season and lowest during the spring (Figure 2). Habitat type influenced bobcat use of an area in the spring and winter, with habitat type included in the top model (S5). Overall, bobcats tended to have the highest use of shelterwood habitat type throughout the year. In spring, the odds of bobcats using shelterwoods was 6.04 times more likely than the use of fields (95% CI = 1.165–12.36) (S6; Figure 2). Habitat type did not impact bobcat use in the summer and fall, as it was not included in the top model (S5). But in winter, again the odds bobcats used shelterwoods was 4.46 times more than fields (95% CI = 1.12–4.62) and 5.803 times more than the use of reserves (95% CI = 1.15–10.55) (S5; Figure 2). Average estimates of coyote use in an area were the highest during the spring season and the lowest during the fall season (Figure 2). Habitat type only influenced coyote use of an area during the spring, with habitat type occurring in the top model (S5). The odds of coyotes using fields was 5.47 times more likely than in reserve habitat (95% CI = 1.06–8.49), and 4.28 times more likely than in the use of shelterwood habitat (95% CI =1.05–4.18). The likelihood between reserve and shelterwood habitats was similar (S6). During summer, fall, and winter, habitat type did not seem to influence coyote use of an area, as habitat type was not included in the top coyote model (S5). Raccoons had the highest average use during the spring season and tended to use fields more than other habitat types (Figure 2). Habitat type impacted raccoons only during one season (the fall), with habitat type not occurring in the top model for the other three seasons (S5). During fall, the odds raccoons using fields was 4.07 times more likely than reserves (95% CI = 1.08–3.74) and 3.9 times more likely than raccoon use of shelterwoods (95% CI = 1.05–3.52). There was no difference in the odds of using reserves and shelterwoods (S6; Figure 2). Overall, fisher occupancy of the YMF landscape was half or less than that of the other three mesocarnivores and tended to use reserve habitats throughout the year (Figure 2). During the spring and fall, habitat type influenced fisher use of an area, occurring in the top model for both seasons (S5). In the spring, the odds of fisher using reserves was 4.68 times more likely than the use of shelterwood (95% CI = 1.09–7.25), and the use of reserve was 5.22 times more likely than fields (95% CI = 1.54–5.12) (S6). In the fall, the odds of fisher using shelterwood was 3.82 times more likely than fishers using fields (95% CI = 1.07–3.30). During the summer and winter, fisher use was not influenced by habitat type.

## 4 DISCUSSION

Our year‐long study highlights the beneficial role that working lands (managed forests and farms) play in maintaining mesopredators in northeastern landscapes. Our results specifically show shelterwood cuts can support mesopredator conservation, in particular for bobcat and fisher. We also found none of the mesopredator species were found to have single, specific habitats that they used exclusively throughout the year. Therefore, in the interest of mesopredator conservation, a mosaic of working lands and reserve forests will likely not diminish mesopredator species presence across the landscape relative to merely protecting intact mature forested landscapes alone. Hence, in this landscape, mesopredator conservation can align with rather than be jeopardized by multiple land uses.

Many occupancies and use studies are only conducted within a single season (Agha et al., 2018). Our multiple season analysis instead responds to the growing recognition that wildlife studies, intended to inform landscape planning for the conservation of mesopredator species, need to be conducted throughout the entire year to account for seasonal variation in use (Atwood et al., 2004; Ikeda et al., 2016; Ray, 2010 Lesmeister et al., 2015; Zieliksi et al. 2015). Our analysis revealed that each species had differential use across habitat types during some of the year, but no difference in the use of habitat types at other times (Figure 2 and S5). In particular, it revealed that mesopredator species used the entire landscape throughout the year, suggestive of tolerance to human land‐use changes when reserve forest habitat is present. But the highest use level of habitat type varied among species, with three of the four species associated with different human‐disturbance. Bobcats tended to use harvested shelterwoods the most throughout the year while fishers were the only species who primarily utilized intact undisturbed forest (Figure 2). Nevertheless, all species appeared to exhibit generalized presence among all land uses throughout the year, affirming the designation of three of the four mesopredator species (coyote, raccoon, and bobcat) as habitat generalists (Owen et al., 2015; Reed et al., 2017; Way et al., 2004). Fisher—while largely a forested habitat specialist, may also show a more generalist tendency, at least with respect to the use of forest harvested using silvicultural methods such as shelterwoods that promote habitat values sought by this species including canopy cover, foliage height diversity, and remnant snags. Though no overdispersion was found and site selection reduced the likelihood of this, if mesopredator home ranges fell within groups of our sites, the potential for nonindependence could inflate the statistical power of our results.

Despite their generalized presence, there were differences in the particular coarse‐ and fine‐scale habitat features with which each species is associated in each of the land uses. Foliage height diversity consistently predicted bobcat use across all seasons, and fisher occupancy in two seasons. In the summer, bobcat use of an area increased further from roads, with raccoon use increasing closer to roads (Table 3). Coyotes and fishers were positively associated with a closed canopy in the spring, whereas bobcats were negatively associated with closed canopy in the winter (Table 3). Snag density also positively influenced fishers during the summer and fall months (S4). With such seasonal variation, considering only single seasonal occupancy would fail to account for time‐varying importance of different coarse‐ and fine‐scale habitat features (S4). Our results also highlight the need to manage both coarse‐ and fine‐scale habitat variables (such as structural diversity and road distance) for habitat conservation.

As a community, the mesopredator species exhibited complementary use patterns among habitats and seasons. Coyotes and raccoons exhibited similar yearlong patterns of habitat use and similar rank‐order of use of the three habitats within each season (Figure 2), whereas bobcats had the opposite trend and rank‐order of habitat use (Figure 2). These differences in use may reflect seasonal differences in habitat needs. The pattern of coyotes being completely habitat generalized (habitat type only impacting coyotes in the spring), with minimal habitat variable associations during fall and winter may reflect an increased movement to use the landscape more broadly in response to the scarcity of small mammal prey base during winter months than in the summer (Cummings & Vessey, 1994; Flowerdew et al., 2017; Way et al., 2004). Fields were more likely to be used by coyotes and raccoons during spring and fall. Such agricultural lands provide a greater abundance of prey, including small mammals and deer during these seasons than do forested habitats (Crete et al., 2001; Hubert et al., 2003; Gosselinke et al., 2003). Fisher exhibited fairly uniform use of reserve and shelterwoods throughout the year. Bobcat occupancy of fields was generally low throughout the year (Figure 2), consistent with previous studies (Bradley and Farge, 1988; Preuss & Gehring, 2007). Bobcat's tendency to have the highest use of shelterwoods may be explained by two factors. First, shelterwoods are more suitable for hares and rabbits—key bobcat prey species—than are fields and mature forest reserves (Orr & Dodds, 1982; Litvaitis et al., 1986, 2006). Second, occupancy patterns may be determined by finer‐scale habitat variables (Reed et al., 2017). Fields generally tend to be closer to public roads than forested habitats (Bled et al., 2015; Fahrig & Rytwinski, 2009) and we found that bobcats were negatively associated with roads (Table 3). Moreover, bobcats were positively associated with habitat structural diversity (Table 3). Recently cut shelterwoods are early successional habitat, providing more diverse structure (foliage height diversity and canopy cover) with ample CWD and dense regeneration. Such structure has a positive impact on small mammal and bird prey populations (Fuller et al., 2003; Goodale et al., 2009; Kailes, 2010; Zwolak, 2009).

Coyote and raccoon use were both lowest in shelterwoods throughout the year (Figure 2). For coyotes, this is even with their potential positive association to skid roads (which are only found in shelterwoods) (Table 3). Similarly, raccoon use was highest in fields, fitting with their positive association with roads in two seasons (Table 2). Unlike bobcats, the habitat variables appear to not strongly influence coyotes or raccoons. Likely, resources and prey densities are more the driving factors of raccoon and coyote occupancy, especially during colder months (Newbury & Nelson, 2007; Patterson & Messier, 2001; Pedlar et al., 1997; Way et al., 2004).

Coyote and bobcat use of reserves were both moderate throughout the year. For bobcats, this is potentially due to reserves generally being farther from roads, as well as, providing necessary denning habitat, including rock features and downed trees (Broman et al., 2014; Litvaitis et al., 1986). For coyotes, this moderate use is in part surprising, given that more mature forests are often thought of as marginal habitats for coyotes providing less prey availability and less efficient hunting (Kays et al., 2008; Thibault & Ouellet, 2005). However, coyotes are consistently found in forest reserve habitats (Crete et al., 2001; Hinton et al., 2015). Raccoons had their lowest occupancies in reserves, being more adapted to human‐altered landscapes (Beasley et al., 2011; Ray, 2000).

Our results show the beneficial impact of fields on certain mesopredator species. With this comes the increased potential for human–coyote or human–raccoon interactions in these habitats. Such potential for conflict is increasing of interest to wildlife and forest managers. Coyotes cost millions of dollars in livestock losses every year and increasing mesopredator populations have been linked to increased incidences of Lyme disease (Levi et al., 2012; Vercauteren et al., 2012). The study also highlights the benefit of timber management for mesopredators, especially bobcats and fishers. These habitats can create forest structural diversity, and increase CWD, in turn, increases small mammal and bird populations (Fuller et al., 2003, Zwolak, 2009, Kailes, 2010). Additionally, skid roads and logging roads provide linear features which may benefit movement through the system by coyotes and other canids (Fisher & Burton, 2018; McKenzie et al., 2012).

Our study emphasizes that the inclusion of early successional forests in the landscape matrix has the potential to increase certain mesopredators species. While altered timber harvest forest can increase the occupancy of certain mesopredators, reserve habitats are also important to maintain multiple mesopredators on the landscape. This highlights the possibility to manage landscapes for a diverse mosaic of agriculture, reserve, and timber harvest without diminishing mesopredator species on the landscape.

### 4.1 Conservation and management implications

Conservationists and wildlife managers often solely focus on/argue for the protection of intact, mature forests to conserve and restore mesopredator and other predator populations. However, this conventional conservation argument may not be accurate (Proulx 2020; Ray 2000). Our work highlights the importance of considering other rural land uses in conservation efforts and shows that protecting only mature forests can overlook and potentially fail to conserve beneficial habitat characteristics for wildlife. Shelterwood cuts and other similar harvests, which increase the structural diversity of the forest and open up the forest canopy, can enhance bobcat conservation. These beneficial characteristics found in shelterwood cuts can be lacking in aging, unmanaged forests. Timber harvest systems also have the potential to support fisher conservation if a high density of snags are left untouched, and reserve forest occurs nearby (Bunnel & Houde, 2010; Degraaf & Yamasaki, 2001; Powell & Zielinski 1994). Instead of focusing on intact forest protection alone, our study ultimately reveals that a mosaic of timber management, smallholder agricultural, and reserve forest can be utilized to conserve mammalian mesopredator diversity on a landscape. Our findings illustrate the need for conservationists, farmers, and foresters to work together to create landscape‐level planning that integrates rural livelihoods and wildlife conservation.

## AUTHOR CONTRIBUTIONS

**Kimberly Zamuda:** Conceptualization (equal); data curation (lead); formal analysis (lead); funding acquisition (lead); investigation (lead); methodology (equal); project administration (lead); resources (lead); software (lead); supervision (lead); validation (equal); visualization (equal); writing – original draft (equal); writing – review and editing (equal). **Marlyse C. Duguid:** Formal analysis (equal); methodology (equal); supervision (supporting); validation (equal); visualization (equal); writing – original draft (supporting); writing – review and editing (equal). **Oswald Schmitz:** Conceptualization (equal); formal analysis (equal); funding acquisition (supporting); methodology (equal); project administration (supporting); supervision (equal); validation (equal); visualization (equal); writing – original draft (equal); writing – review and editing (equal).

## CONFLICT OF INTEREST

The authors declare no conflict of interest.

## Supporting information


Appendix S1

## Data Availability

The data from this study can be found in Dryad Digital Repository. https://doi.org/10.5061/dryad.7d7wm37xs.
