# Supplementary material for: Human land‐use effects on mammalian mesopredator occupancy of a northeastern Connecticut landscape
Source: Ecol Evol. 2022 Jul 3;12(7):e9015. doi: 10.1002/ece3.9015 (PMC9251285; doi:10.1002/ece3.9015)
Supplement: Supplementary file 1 — Appendix S1 [file ECE3-12-e9015-s001.docx]

S1. Study Focus species and non-focus species list

| **Species Name** | **Common Name** |
| --- | --- |
| *Canis latrans* | Coyote |
| *Lynx rufus* | Bobcat |
| *Procyon lotor* | Raccoon |
| *Penkania pennanti* | Fisher |
| *Vulpes Vulpes* | Red fox |
| *Urocyon cinereoargenteus* | Grey fox |
| *Mephitis mephitis* | Striped skunk |
| *Didelphis virginiana* | Virginia opossum |
| *Odocoileus virginianus* | White tailed deer |
| *Sciurus carolinensis* | Eastern gray squirrel |
| *Glaucomys volans* | Flying squirrel |
| *Lontra canadensis* | River Otter |
| *Buteo jamaicensis* | Red-tailed hawk |
| *Meleagris gallopavo* | Wild turkey |
| *Strix varia* | Barred owl |
| *Neovison vison* | American mink |
| *Sylvilagus floridanus* | Eastern cottontail |
| Peromyscus spp | mouse |
| -- | Various bird species |

S2.

Imposed over the study area map, the zoomed-in map illustrates the relocation of camera traps between sites during the spring season. Three reserve (circle), three shelterwood (square) and two field (triangle) camera trapping sites are highlighted. For set one, we placed the cameras at Site A, D and G for three weeks. For set two, we moved the cameras to Site B, E and H. For set three, we moved the cameras to a new reserve site (Site C), a new shelterwood site (Site F), and returned the field cameras to Site G (not illustrated). This process was identical for all four seasons.

S3. Habitat variable occupancy model selection for mesopredator species in and around Yale Myers Forest, Connecticut, USA. The detection covariates included in each model were from the best (most parsimonious) model determined by the detection probability model selection for each species.

| Model^a^ | AIC^b^ | ΔAIC | *w*^c^ | K^d^ | -2LogLike^f^ |  |
| --- | --- | --- | --- | --- | --- | --- |
|  |  |  |  |  |  |  |
| *Bobcat, spring* | | | | | |  |
| FHD | 559.20 | 0.00 | 0.487 | 4 | 551.2 |  |
| FHD + DPR | 561.48 | 2.28 | 0.206 | 5 | 551.48 |  |
| CC +FHD | 561.76 | 2.56 | 0.119 | 5 | 551.76 |  |
| FHD + DPR + CC | 562.81 | 3.61 | 0.069 | 6 | 550.81 |  |
| CC + DSR +DPR | 564.19 | 4.99 | 0.028 | 6 | 552.19 |  |
| CC | 565.43 | 6.23 | 0.019 | 4 | 557.43 |  |
| DRS +CC +FHD | 566.64 | 7.44 | 0.008 | 6 | 554.64 |  |
| DPR | 566.88 | 7.68 | 0.008 | 4 | 558.88 |  |
| FHD +CC +DPR + DSR | 567.10 | 7.90 | 0.008 | 7 | 553.1 |  |
| DRS + FHD | 567.81 | 8.61 | 0.004 | 5 | 557.81 |  |
| *(.)* | 567.95 | 8.75 | 0.004 | 3 | 561.95 |  |
| CC + DSR | 568.21 | 9.01 | 0.002 | 5 | 558.21 |  |
| DSR + DPR | 568.42 | 9.22 | 0.002 | 5 | 558.42 |  |
| DSR | 568.76 | 9.56 | 0.002 | 4 | 560.76 |  |
| DPR + CC | 568.95 | 9.75 | 0.002 | 5 | 558.95 |  |
| *Bobcat, summer* | | | | | |  |
| FHD + DPR | 510.2 | 0 | 0.384 | 5 | 500.2 |  |
| FHD | 510.36 | 0.16 | 0.331 | 4 | 502.36 |  |
| FHD + DPR + CC | 511.74 | 1.54 | 0.183 | 6 | 499.74 |  |
| FHD +CC | 512.72 | 2.52 | 0.050 | 5 | 502.72 |  |
| DPR + CC | 513.13 | 2.93 | 0.036 | 5 | 503.13 |  |
| DPR | 515.97 | 5.77 | 0.009 | 4 | 507.97 |  |
| CC | 516.32 | 6.12 | 0.004 | 4 | 508.32 |  |
| FHD + DSR | 517.11 | 6.91 | 0.003 | 5 | 507.11 |  |
| FHD +CC +DPR + DSR | 518.08 | 7.88 | 0.000 | 7 | 504.08 |  |
| (.) | 519.41 | 9.21 | 0.000 | 3 | 513.41 |  |
| DPR + DSR | 519.54 | 9.34 | 0.000 | 5 | 509.54 |  |
| CC + DSR | 519.7 | 9.5 | 0.000 | 5 | 509.7 |  |
| DSR | 520.4 | 10.2 | 0.000 | 4 | 512.4 |  |
| *Bobcat, fall* | | | | | |  |
| FHD | 523.1 | 0 | 0.53 | 4 | 515.1 |  |
| DRS + FHD | 525.6 | 2.5 | 0.21 | 5 | 515.6 |  |
| DRS +CC +FHD | 526.22 | 3.12 | 0.102 | 6 | 514.22 |  |
| FHD + DPR | 528.42 | 5.32 | 0.043 | 5 | 518.42 |  |
| FHD + DPR + CC | 529.11 | 6.01 | 0.01 | 6 | 517.11 |  |
| CC | 529.19 | 6.09 | 0.009 | 4 | 521.19 |  |
| FHD +CC +DPR + DSR | 530.33 | 7.23 | 0.005 | 7 | 516.33 |  |
| FHD +CC | 530.44 | 7.34 | 0.003 | 5 | 520.44 |  |
| DSR | 530.55 | 7.45 | 0.003 | 4 | 522.55 |  |
| CC + DSR | 531.6 | 8.5 | 0.002 | 5 | 521.6 |  |
| DPR + CC | 532.2 | 9.1 | 0.002 | 5 | 522.2 |  |
| DSR + DPR | 532.97 | 9.87 | 0.002 | 5 | 522.97 |  |
| DPR | 534.53 | 11.43 | 0.001 | 4 | 526.53 |  |
| CC + DSR +DPR | 535.02 | 11.92 | 0.001 | 6 | 523.02 |  |
| (.) | 535.64 | 12.54 | 0.001 | 3 | 529.64 |  |
| *Bobcat, winter* | | | | | |  |
| FHD | 499.6 | 0 | 0.335 | 4 | 491.6 |  |
| FHD + CC | 500.38 | 0.78 | 0.319 | 5 | 490.38 |  |
| DPR + FHD | 501.57 | 1.97 | 0.128 | 5 | 491.57 |  |
| FHD + DPR + CC | 502.84 | 3.24 | 0.091 | 6 | 490.84 |  |
| CC | 503.83 | 4.23 | 0.09 | 4 | 495.83 |  |
| DPR + CC | 507.45 | 7.85 | 0.007 | 5 | 497.45 |  |
| FHD +CC +DPR + DSR | 508.14 | 8.54 | 0.004 | 7 | 494.14 |  |
| CC + DSR | 508.18 | 8.58 | 0.004 | 5 | 498.18 |  |
| FHD + DSR | 509.53 | 9.93 | 0.002 | 5 | 499.53 |  |
| CC + DSR +DPR | 509.9 | 10.3 | 0.002 | 6 | 497.9 |  |
| DRS + CC +FHD | 510.47 | 10.87 | 0.001 | 6 | 498.47 |  |
| DPR + DSR | 511.78 | 12.18 | 0.001 | 5 | 501.78 |  |
| (.) | 513.24 | 13.64 | 0.001 | 3 | 507.24 |  |
| DPR | 513.8 | 14.2 | 0.001 | 4 | 505.8 |  |
| DAR | 514.2 | 14.6 | 0.001 | 4 | 506.2 |  |
| *Coyote, spring* | | | | | |  |
| CC | 724.87 | 0 | 0.467 | 3 | 718.87 |  |
| CC + DPR | 726.74 | 1.87 | 0.255 | 4 | 718.74 |  |
| FOR + CC +DPR | 728.1 | 3.23 | 0.147 | 5 | 718.1 |  |
| FOR + CC | 728.91 | 4.04 | 0.089 | 4 | 720.91 |  |
| CC + DSR +DPR | 730.79 | 5.92 | 0.009 | 5 | 720.79 |  |
| DPR | 731.22 | 6.35 | 0.008 | 3 | 725.22 |  |
| DSR | 732.52 | 7.65 | 0.006 | 3 | 726.52 |  |
| DPR + FOR | 732.81 | 7.94 | 0.006 | 4 | 724.81 |  |
| DSR + CC | 733.44 | 8.57 | 0.006 | 4 | 725.44 |  |
| CC + DPR + DSK +FOR | 734.34 | 9.47 | 0.002 | 6 | 722.34 |  |
| DPR +DSR | 734.9 | 10.03 | 0.002 | 4 | 726.9 |  |
| FOR | 735.86 | 10.99 | 0.001 | 3 | 729.86 |  |
| FOR + DPR + DSK | 736.54 | 11.67 | 0.001 | 5 | 726.54 |  |
| (.) | 737.19 | 12.32 | 0.001 | 2 | 733.19 |  |
| FOR + DSR | 738.38 | 13.51 | 0.000 | 4 | 730.38 |  |
| *Coyote, summer* | | | | | |  |
| DSR + CC | 756.33 | 0 | 0.432 | 5 | 746.33 |  |
| CC | 756.78 | 0.45 | 0.404 | 4 | 748.78 |  |
| FOR + CC | 758.67 | 2.34 | 0.103 | 5 | 748.67 |  |
| FOR + CC +DSR | 759.28 | 2.95 | 0.013 | 6 | 747.28 |  |
| DSR | 760.9 | 4.57 | 0.009 | 4 | 752.9 |  |
| DPR + FOR | 761.26 | 4.93 | 0.006 | 5 | 751.26 |  |
| FOR + DSR | 761.29 | 4.96 | 0.006 | 5 | 751.29 |  |
| CC +DPR | 761.45 | 5.12 | 0.006 | 5 | 751.45 |  |
| FOR | 762.67 | 6.34 | 0.003 | 4 | 754.67 |  |
| CC + DPR + DSK +FOR | 762.81 | 6.48 | 0.002 | 7 | 748.81 |  |
| DPR | 762.96 | 6.63 | 0.002 | 4 | 754.96 |  |
| DPR +DSR | 763.35 | 7.02 | 0.002 | 5 | 753.35 |  |
| CC + DSR +DPR | 764.12 | 7.79 | 0.001 | 6 | 752.12 |  |
| (.) | 765.55 | 9.22 | 0.001 | 3 | 759.55 |  |
| FOR + DPR + DSK | 766.58 | 10.25 | 0.001 | 5 | 756.58 |  |
| *Coyote, fall* | | | | | |  |
| FOR + CC +DSR | 710.24 | 0 | 0.254 | 6 | 698.24 |  |
| CC | 711.18 | 0.94 | 0.197 | 4 | 703.18 |  |
| (.) | 711.56 | 1.32 | 0.188 | 3 | 705.56 |  |
| FOR + CC | 712.36 | 2.12 | 0.105 | 5 | 702.36 |  |
| DSR | 713.19 | 2.95 | 0.098 | 4 | 705.19 |  |
| DPR + FOR | 713.8 | 3.56 | 0.067 | 5 | 703.8 |  |
| DPR | 714.91 | 4.67 | 0.034 | 4 | 706.91 |  |
| CC + DPR + DSK +CC | 716.14 | 5.9 | 0.016 | 5 | 706.14 |  |
| CC +DPR | 717.01 | 6.77 | 0.009 | 5 | 707.01 |  |
| DSR + CC | 717.12 | 6.88 | 0.006 | 5 | 707.12 |  |
| FOR | 717.26 | 7.02 | 0.005 | 4 | 709.26 |  |
| DPR +DSR | 717.45 | 7.21 | 0.004 | 5 | 707.45 |  |
| CC + DSR +DPR | 718.02 | 7.78 | 0.004 | 6 | 706.02 |  |
| FOR + DPR + DSK | 718.25 | 8.01 | 0.002 | 6 | 706.25 |  |
| FOR + DSR | 719.48 | 9.24 | 0.002 | 7 | 705.48 |  |
| *Coyote, winter* | | | | | |  |
| (.) | 742.36 | 0 | 0.249 | 3 | 736.36 |  |
| FOR + CC | 744.43 | 2.07 | 0.195 | 5 | 734.43 |  |
| FOR + CC +DPR | 744.7 | 2.34 | 0.142 | 6 | 732.7 |  |
| CC +DPR | 745.04 | 2.68 | 0.119 | 4 | 737.04 |  |
| CC | 745.19 | 2.83 | 0.097 | 4 | 737.19 |  |
| DSR | 745.35 | 2.99 | 0.085 | 4 | 737.35 |  |
| DPR + FOR | 745.39 | 3.03 | 0.045 | 5 | 735.39 |  |
| FOR + DSR | 745.57 | 3.21 | 0.023 | 5 | 735.57 |  |
| FOR | 745.62 | 3.26 | 0.009 | 4 | 737.62 |  |
| DSR + CC | 745.67 | 3.31 | 0.009 | 5 | 735.67 |  |
| CC + DSR +DPR | 745.81 | 3.45 | 0.006 | 6 | 733.81 |  |
| DPR +DSR | 746 | 3.64 | 0.006 | 5 | 736 |  |
| DPR | 746.03 | 3.67 | 0.006 | 4 | 738.03 |  |
| FOR + DPR + DSK | 746.34 | 3.98 | 0.005 | 6 | 734.34 |  |
| CC + DPR + DSK +CC | 746.86 | 4.5 | 0.004 | 7 | 732.86 |  |
| *Fisher, spring* | | | | | |  |
| CC + FHD | 325.94 | 0 | 0.301 | 8 | 309.94 |  |
| CC | 326.15 | 0.21 | 0.256 | 7 | 312.15 |  |
| FHD | 326.51 | 0.57 | 0.224 | 7 | 312.51 |  |
| CC + FHD + SD | 328.1 | 2.16 | 0.127 | 9 | 310.1 |  |
| SD + FHD | 328.19 | 2.25 | 0.056 | 8 | 312.19 |  |
| CC+ SD | 328.72 | 2.78 | 0.017 | 8 | 312.72 |  |
| CC + FHD + CWD +SD | 328.88 | 2.94 | 0.006 | 10 | 308.88 |  |
| SD | 328.91 | 2.97 | 0.005 | 7 | 314.91 |  |
| CC+CWD | 329.09 | 3.15 | 0.002 | 8 | 313.09 |  |
| (.) | 329.16 | 3.22 | 0.002 | 6 | 317.16 |  |
| FHD +CWD | 329.44 | 3.5 | 0.001 | 8 | 313.44 |  |
| SD + CWD | 329.63 | 3.69 | 0.001 | 8 | 313.63 |  |
| CWD | 329.69 | 3.75 | 0.001 | 7 | 315.69 |  |
| FHD +CWD +SD | 329.77 | 3.83 | 0.001 | 9 | 311.77 |  |
| SD + CC + CWD | 329.9 | 3.96 | 0.000 | 9 | 311.9 |  |
| *Fisher, summer* | | | | | |  |
| SD | 305.88 | 0 | 0.374 | 4 | 297.88 |  |
| SD +FHD | 306.33 | 0.45 | 0.328 | 5 | 296.33 |  |
| FHD | 307.93 | 2.05 | 0.091 | 4 | 299.93 |  |
| CC + FHD + SD | 308 | 2.12 | 0.082 | 6 | 296 |  |
| CC+ SD | 308.25 | 2.37 | 0.053 | 5 | 298.25 |  |
| CC + FHD | 309.29 | 3.41 | 0.023 | 5 | 299.29 |  |
| SD + CWD | 309.57 | 3.69 | 0.019 | 5 | 299.57 |  |
| FHD +CWD +SD | 308.75 | 2.87 | 0.010 | 6 | 296.75 |  |
| CC | 308.89 | 3.01 | 0.009 | 4 | 300.89 |  |
| FHD +CWD | 309.16 | 3.28 | 0.005 | 5 | 299.16 |  |
| CC + FHD + CWD +SD | 309.42 | 3.54 | 0.004 | 7 | 295.42 |  |
| CC+CWD | 309.98 | 4.1 | 0.001 | 5 | 299.98 |  |
| SD + CC + CWD | 310.11 | 4.23 | 0.001 | 6 | 298.11 |  |
| (.) | 310.44 | 4.56 | 0.000 | 3 | 304.44 |  |
| CWD | 310.63 | 4.75 | 0.000 | 4 | 302.63 |  |
| *Fisher, fall* | | | | | |  |
| SD | 312.34 | 0 | 0.523 | 4 | 304.34 |  |
| CC+ SD | 313.98 | 1.64 | 0.287 | 5 | 303.98 |  |
| SD +FHD | 314.47 | 2.13 | 0.053 | 5 | 304.47 |  |
| CC + FHD + SD | 315.1 | 2.76 | 0.042 | 6 | 303.1 |  |
| CC | 315.75 | 3.41 | 0.036 | 4 | 307.75 |  |
| CC + FHD | 316.21 | 3.87 | 0.021 | 5 | 306.21 |  |
| FHD | 316.57 | 4.23 | 0.013 | 4 | 308.57 |  |
| SD + CWD | 317.59 | 5.25 | 0.007 | 5 | 307.59 |  |
| CWD | 318.13 | 5.79 | 0.005 | 4 | 310.13 |  |
| FHD +CWD +SD | 319.18 | 6.84 | 0.003 | 6 | 307.18 |  |
| CC+CWD | 319.67 | 7.33 | 0.002 | 5 | 309.67 |  |
| (.) | 320.52 | 8.18 | 0.002 | 3 | 314.52 |  |
| FHD +CWD | 321.91 | 9.57 | 0.002 | 5 | 311.91 |  |
| SD + CC + CWD | 322.77 | 10.43 | 0.002 | 6 | 310.77 |  |
| CC + FHD + CWD +SD | 323.38 | 11.04 | 0.002 | 7 | 309.38 |  |
| *Fisher, winter* | | | | | |  |
| FHD + SD | 348.23 | 0 | 0.287 | 6 | 336.23 |  |
| FHD | 348.61 | 0.38 | 0.259 | 5 | 338.61 |  |
| CC +FHD + SD | 351.37 | 3.14 | 0.098 | 7 | 337.37 |  |
| CC + FHD | 351.55 | 3.32 | 0.09 | 6 | 339.55 |  |
| SD | 351.8 | 3.57 | 0.082 | 5 | 341.8 |  |
| CC+ SD | 352.17 | 3.94 | 0.065 | 6 | 340.17 |  |
| FHD +CWD | 352.89 | 4.66 | 0.042 | 6 | 340.89 |  |
| CC | 353.36 | 5.13 | 0.021 | 5 | 343.36 |  |
| SD + CWD | 354.14 | 5.91 | 0.018 | 6 | 342.14 |  |
| CWD | 354.66 | 6.43 | 0.009 | 5 | 344.66 |  |
| FHD +CWD +SD | 355.1 | 6.87 | 0.007 | 7 | 341.1 |  |
| CC+CWD | 355.15 | 6.92 | 0.006 | 6 | 343.15 |  |
| CC + FHD + CWD +SD | 355.37 | 7.14 | 0.006 | 8 | 339.37 |  |
| SD + CC + CWD | 355.52 | 7.29 | 0.005 | 7 | 341.52 |  |
| (.) | 355.59 | 7.36 | 0.005 | 4 | 347.59 |  |
| *Raccoon, spring* | | | | | |  |
| WET | 274.92 | 0 | 0.243 | 4 | 266.92 |  |
| DPR + WET | 276.12 | 1.2 | 0.181 | 5 | 266.12 |  |
| WET + FOR | 277.66 | 2.74 | 0.081 | 5 | 267.66 |  |
| DPR + WET + FOR | 277.74 | 2.82 | 0.078 | 6 | 265.74 |  |
| SD+WET | 278.51 | 3.59 | 0.073 | 5 | 268.51 |  |
| WET + SD +DPR | 278.63 | 3.71 | 0.069 | 6 | 266.63 |  |
| DPR | 278.8 | 3.88 | 0.067 | 4 | 270.8 |  |
| FOR | 278.99 | 4.07 | 0.064 | 4 | 270.99 |  |
| SD+ DPR | 279.14 | 4.22 | 0.056 | 5 | 269.14 |  |
| DPR + FOR | 279.28 | 4.36 | 0.040 | 5 | 269.28 |  |
| SD | 279.85 | 4.93 | 0.028 | 4 | 271.85 |  |
| SD + FOR | 279.95 | 5.03 | 0.008 | 5 | 269.95 |  |
| WET +FOR + SD + DPR | 280.06 | 5.14 | 0.006 | 7 | 266.06 |  |
| (.) | 280.2 | 5.28 | 0.006 | 3 | 274.2 |  |
| *Raccoon, summer* | | | | | |  |
| DPR | 268.33 | 0 | 0.278 | 4 | 260.33 |  |
| DPR + WET + FOR | 269.36 | 1.03 | 0.216 | 6 | 257.36 |  |
| DPR + WET | 269.9 | 1.57 | 0.171 | 5 | 259.9 |  |
| WET | 270.38 | 2.05 | 0.077 | 4 | 262.38 |  |
| WET +FOR + SD + DPR | 270.72 | 2.39 | 0.048 | 7 | 256.72 |  |
| DPR + FOR | 271.2 | 2.87 | 0.040 | 5 | 261.2 |  |
| WET + SD +DPR | 271.24 | 2.91 | 0.035 | 6 | 259.24 |  |
| SD+ DPR | 271.38 | 3.05 | 0.030 | 5 | 261.38 |  |
| WET + FOR | 271.42 | 3.09 | 0.027 | 5 | 261.42 |  |
| SD | 271.59 | 3.26 | 0.023 | 4 | 263.59 |  |
| (.) | 271.63 | 3.3 | 0.021 | 3 | 265.63 |  |
| SD+WET | 271.92 | 3.59 | 0.015 | 5 | 261.92 |  |
| SD + FOR | 271.93 | 3.6 | 0.010 | 5 | 261.93 |  |
| FOR | 271.95 | 3.62 | 0.009 | 4 | 263.95 |  |
| *Raccoon, fall* | | | | | |  |
| FOR | 250.24 | 0 | 0.192 | 4 | 242.24 |  |
| FOR +WET | 252.07 | 1.83 | 0.157 | 5 | 242.07 |  |
| (.) | 252.31 | 2.07 | 0.094 | 3 | 246.31 |  |
| DPR + FOR | 252.79 | 2.55 | 0.089 | 5 | 242.79 |  |
| SD + FOR | 253.08 | 2.84 | 0.087 | 5 | 243.08 |  |
| SD + WET + FOR | 253.41 | 3.17 | 0.074 | 6 | 241.41 |  |
| DPR + WET | 254.73 | 4.49 | 0.061 | 5 | 244.73 |  |
| WET | 255.28 | 5.04 | 0.044 | 4 | 247.28 |  |
| SD+WET | 255.34 | 5.1 | 0.041 | 5 | 245.34 |  |
| WET +FOR + SD + DPR | 255.49 | 5.25 | 0.039 | 7 | 241.49 |  |
| DPR | 255.97 | 5.73 | 0.036 | 4 | 247.97 |  |
| SD+ DPR | 256.42 | 6.18 | 0.031 | 5 | 246.42 |  |
| SD | 256.53 | 6.29 | 0.028 | 4 | 248.53 |  |
| WET + SD +DPR | 256.55 | 6.31 | 0.027 | 6 | 244.55 |  |
| *Raccoon, winter* | | | | | |  |
| DPR + WET | 285.75 | 0 | 0.210 | 5 | 275.75 |  |
| (.) | 286.01 | 0.26 | 0.196 | 3 | 280.01 |  |
| DPR + WET + FOR | 286.72 | 0.97 | 0.118 | 6 | 274.72 |  |
| DPR | 287.89 | 2.14 | 0.066 | 4 | 279.89 |  |
| WET | 288.01 | 2.26 | 0.058 | 4 | 280.01 |  |
| WET + SD +DPR | 288.07 | 2.32 | 0.055 | 6 | 276.07 |  |
| SD+WET | 288.16 | 2.41 | 0.051 | 5 | 278.16 |  |
| DPR + FOR | 288.31 | 2.56 | 0.042 | 5 | 278.31 |  |
| SD+ DPR | 288.74 | 2.99 | 0.041 | 5 | 278.74 |  |
| FOR | 289.12 | 3.37 | 0.039 | 4 | 281.12 |  |
| SD + FOR | 289.47 | 3.72 | 0.035 | 5 | 279.47 |  |
| WET + FOR | 288.81 | 3.06 | 0.035 | 5 | 278.81 |  |
| WET +FOR + SD + DPR | 289.93 | 4.18 | 0.029 | 7 | 275.93 |  |
| SD | 290.02 | 4.27 | 0.025 | 4 | 282.02 |  |

^a^ FHD, foliage height diversity; DPR, distance to public road; CC, canopy cover; FOR, percent forest; DSR, distance to skid road; SD, snag density; CWD, coarse woody debris, WET, percent wetland,

^b^ Difference in Akaike’s Information Criterion from the top model to current model

^c^ Model weight (model probability)

^d^ Number of model parameters

^e^ -2Log(Likelihood), measure of model fit

S4. All estimated coefficients for habitat variables by species and season from our top ranked occupancy models in the managed Yale-Myers Research Forest, located in northeastern Connecticut (with SE). We considered coefficients significant when the 95% confidence interval did not overlap with 0.

| Species | | Season | | Variable | Estimated coefficients | | SE | LCI | UCI |
| --- | --- | --- | --- | --- | --- | --- | --- | --- | --- |
| Bobcat | Spring | | Intercept | | | -0.759 | 0.371 | -1.486 | -0.032 |
|  |  | | Canopy Cover | | | -0.035 | 0.320 | -0.663 | 0.593 |
|  |  | | Foliage Height Diversity | | | 0.352 | 0.102 | 0.152 | 0.552 |
|  |  | | Distance to Public Road | | | 0.472 | 0.394 | -1.243 | 0.300 |
|  |  | | Distance to Stream or River | | | -0.149 | 0.290 | -0.717 | 0.420 |
|  |  | |  | | |  |  |  |  |
|  | Summer | | Intercept | | | -0.253 | 0.565 | -1.360 | 0.854 |
|  |  | | Canopy Cover | | | 0.512 | 0.269 | -0.015 | 1.039 |
|  |  | | Foliage Height Diversity | | | 0.923 | 0.186 | 0.559 | 1.287 |
|  |  | | Distance to Public Road | | | 0.925 | 0.401 | 0.139 | 1.711 |
|  |  | | Distance to Stream or River | | | -0.058 | 0.419 | -0.878 | 0.763 |
|  |  | |  | | |  |  |  |  |
|  | Fall | | Intercept | | | 1.190 | 1.935 | -2.603 | 4.983 |
|  |  | | Canopy Cover | | | 0.267 | 0.439 | -0.593 | 1.127 |
|  |  | | Foliage Height Diversity | | | 0.623 | 0.186 | 0.259 | 0.987 |
|  |  | | Distance to Public Road | | | -0.019 | 0.361 | -0.727 | 0.688 |
|  |  | | Distance to Stream or River | | | -0.428 | 0.247 | -0.912 | 0.056 |
|  |  | |  | | |  |  |  |  |
|  | Winter | | Intercept | | | 0.043 | 0.519 | -0.975 | 1.060 |
|  |  | | Canopy Cover | | | -0.507 | 0.203 | -0.905 | -0.109 |
|  |  | | Foliage Height Diversity | | | 0.739 | 0.356 | 0.041 | 1.437 |
|  |  | | Distance to Public Road | | | 0.009 | 0.290 | -0.559 | 0.577 |
|  |  | | Distance to Stream or River | | | 0.038 | 0.211 | -0.748 | 0.864 |
|  |  | |  | | |  |  |  |  |
| Coyote | Spring | | Intercept | | | 2.502 | 1.701 | -0.831 | 5.835 |
|  |  | | Percent forest | | | -1.520 | 0.090 | -3.284 | 0.244 |
|  |  | | Canopy Cover | | | 0.744 | 0.305 | 0.146 | 1.342 |
|  |  | | Distance to Public Road | | | -0.205 | 0.156 | -0.510 | 0.101 |
|  |  | | Distance to Skid Road | | | -0.038 | 0.667 | -1.345 | 1.270 |
|  |  | |  | | |  |  |  |  |
|  | Summer | | Intercept | | | 0.938 | 0.551 | -0.142 | 2.018 |
|  |  | | Percent forest | | | 0.219 | 0.365 | -0.495 | 0.934 |
|  |  | | Canopy Cover | | | 0.956 | 0.391 | 0.190 | 1.721 |
|  |  | | Distance to Public Road | | | -0.209 | 0.308 | -0.814 | 0.395 |
|  |  | | Distance to Skid Road | | | -0.866 | 0.450 | -1.748 | 0.016 |
|  |  | |  | | |  |  |  |  |
|  | Fall | | Intercept | | | 1.170 | 0.429 | 0.329 | 2.011 |
|  |  | | Percent forest | | | -0.101 | 0.344 | -0.776 | 0.573 |
|  |  | | Canopy Cover | | | 0.208 | 0.348 | -0.474 | 0.890 |
|  |  | | Distance to Public Road | | | -0.115 | 0.322 | -0.746 | 0.516 |
|  |  | | Distance to Skid Road | | | 0.171 | 0.188 | -0.197 | 0.640 |
|  |  | |  | | |  |  |  |  |
|  | Winter | | Intercept | | | 1.174 | 0.425 | 0.342 | 2.006 |
|  |  | | Percent forest | | | -0.866 | 0.685 | -2.209 | 0.477 |
|  |  | | Canopy Cover | | | -0.108 | 0.205 | -0.509 | 0.293 |
|  |  | | Distance to Public Road | | | 0.758 | 0.440 | -0.103 | 1.620 |
|  |  | | Distance to Skid Road | | | -0.134 | 0.370 | -0.592 | 0.860 |
|  |  | |  | | |  |  |  |  |
| Fisher | Spring | | Intercept | | | -2.150 | 0.619 | -3.363 | -0.937 |
|  |  | | Canopy Cover | | | -0.907 | 0.522 | -1.930 | 0.117 |
|  |  | | Foliage Height Diversity | | | -0.530 | 0.259 | -1.038 | -0.022 |
|  |  | | Snag Density | | | 0.635 | 0.295 | 0.056 | 1.213 |
|  |  | |  | | |  |  |  |  |
|  | Summer | | Intercept | | | -0.343 | 1.539 | -3.359 | 2.673 |
|  |  | | Canopy Cover | | | 0.405 | 0.806 | -1.174 | 1.984 |
|  |  | | Foliage Height Diversity | | | 2.460 | 1.200 | 0.108 | 4.812 |
|  |  | | Snag Density | | | 0.654 | 0.329 | 0.010 | 1.298 |
|  |  | |  | | |  |  |  |  |
|  | Fall | | Intercept | | | 1.147 | 0.750 | -0.324 | 2.617 |
|  |  | | Canopy Cover | | | -0.059 | 0.528 | -1.094 | 0.976 |
|  |  | | Foliage Height Diversity | | | 0.070 | 0.357 | -0.629 | 0.769 |
|  |  | | Snag Density | | | 0.928 | 0.450 | 0.046 | 1.810 |
|  |  | |  | | |  |  |  |  |
|  | Winter | | Intercept | | | -1.588 | 0.604 | -2.772 | -0.404 |
|  |  | | Canopy Cover | | | 0.346 | 0.514 | -0.661 | 1.353 |
|  |  | | Foliage Height Diversity | | | 0.493 | 0.200 | 0.101 | 0.885 |
|  |  | | Snag Density | | | 0.242 | 0.340 | -0.424 | 0.908 |
|  |  | |  | | |  |  |  |  |
| Raccoon | Spring | | Intercept | | | 1.081 | 0.513 | 0.075 | 2.087 |
|  |  | | Distance To Public Road | | | -0.577 | 0.320 | -1.204 | 0.049 |
|  |  | | Percent Wetland | | | 0.473 | 0.208 | 0.065 | 0.881 |
|  |  | | Percent Forest | | | -1.681 | 0.920 | -3.484 | 0.122 |
|  |  | |  | | |  |  |  |  |
|  | Summer | | Intercept | | | 0.387 | 0.319 | -0.238 | 1.012 |
|  |  | | Distance To Public Road | | | -0.240 | 0.102 | -0.440 | -0.040 |
|  |  | | Percent Wetland | | | 0.173 | 0.267 | -0.351 | 0.696 |
|  |  | | Percent Forest | | | -0.160 | 0.398 | -0.940 | 0.620 |
|  |  | |  | | |  |  |  |  |
|  | Fall | | Intercept | | | -0.869 | 0.356 | -1.566 | -0.172 |
|  |  | | Distance To Public Road | | | 0.389 | 0.397 | -0.389 | 1.167 |
|  |  | | Percent Wetland | | | 0.489 | 0.414 | -0.423 | 1.200 |
|  |  | | Percent Forest | | | -1.169 | 0.442 | -2.035 | -0.303 |
|  |  | |  | | |  |  |  |  |
|  | Winter | | Intercept | | | -0.486 | 0.450 | -1.368 | 0.396 |
|  |  | | Distance To Public Road | | | 0.172 | 0.195 | -0.2015 | 0.358 |
|  |  | | Percent Wetland | | | 0.929 | 0.571 | -0.190 | 2.048 |
|  |  | | Percent Forest | | | -1.012 | 0.559 | -2.107 | 0.084 |

S5. Habitat type model selection to determine the influence of habitat type on mesopredator species in and around Yale Myers Forest, Connecticut, USA. Habitat type was compared to the top habitat variable model for each species and season (see Table 2). The detection covariates included in each model were from the best (most parsimonious) model for each species determined by model selection.

| Model^a^ | AIC^b^ | ΔAIC | *w*^c^ | K^d^ | -2LogLike^f^ |  |
| --- | --- | --- | --- | --- | --- | --- |
|  |  |  |  |  |  |  |
| *Bobcat: Spring* | | | | | |  |
| Habitat Type + FHD | 558.84 | 0 | 0.371 | 7 | 544.84 |  |
| FHD | 559.2 | 0.36 | 0.346 | 4 | 551.2 |  |
| Habitat Type | 559.72 | 0.88 | 0.283 | 3 | 553.72 |  |
| *Bobcat: Summer* | | | | | |  |
| FHD +DPR | 510.2 | 0 | 0.754 | 5 | 500.2 |  |
| Habitat Type + FHD +DPR | 512.7 | 2.5 | 0.149 | 8 | 496.7 |  |
| Habitat Type | 513.52 | 3.32 | 0.097 | 3 | 507.52 |  |
| *Bobcat: Fall* | | | | | |  |
| FHD | 523.1 | 0 | 0.612 | 4 | 515.1 |  |
| Habitat Type + FHD | 525.17 | 2.07 | 0.259 | 7 | 511.17 |  |
| Habitat Type | 526.46 | 3.36 | 0.129 | 3 | 520.46 |  |
| *Bobcat: Winter* | | | | | |  |
| Habitat Type | 497.61 | 0 | 0.529 | 3 | 491.61 |  |
| Habitat Type + FHD | 498.58 | 0.97 | 0.278 | 7 | 484.58 |  |
| FHD | 499.6 | 1.99 | 0.193 | 4 | 491.6 |  |
| *Coyote: Spring* | | | | | |  |
| Habitat Type | 720.14 | 0 | 0.582 | 3 | 714.14 |  |
| Habitat Type + CC | 721.76 | 1.62 | 0.253 | 6 | 709.76 |  |
| CC | 722.08 | 1.94 | 0.171 | 3 | 716.08 |  |
| *Coyote: Summer* | | | | | |  |
| DSR + CC | 756.33 | 0 | 0.527 | 5 | 746.33 |  |
| Habitat Type + DSR + CC | 758.28 | 1.95 | 0.348 | 8 | 742.28 |  |
| Habitat Type | 758.37 | 2.04 | 0.106 | 3 | 752.37 |  |
| *Coyote: Fall* | | | | | |  |
| FOR + CC + DSR | 710.24 | 0 | 0.656 | 6 | 698.24 |  |
| FOR + CC + DSR + Habitat Type | 712.62 | 2.38 | 0.19 | 9 | 694.62 |  |
| Habitat Type | 713.75 | 3.51 | 0.059 | 3 | 707.75 |  |
| *Coyote: Winter* | | | | | |  |
| (.) | 742.36 | 0 | 0.68 | 3 | 736.36 |  |
| Habitat Type | 745.56 | 3.2 | 0.32 | 3 | 739.56 |  |
| *Fisher: Spring* | | | | | |  |
| Habitat Type | 324.36 | 0 | 0.607 | 3 | 309.94 |  |
| Habitat Type +CC +FHD | 324.9 | 0.54 | 0.235 | 11 | 302.9 |  |
| CC +FHD | 325.94 | 1.58 | 0.158 | 8 | 309.94 |  |
| *Fisher: Summer* | | | | | |  |
| SD | 305.88 | 0 | 0.657 | 4 | 297.88 |  |
| Habitat Type +SD | 307.8 | 1.92 | 0.228 | 7 | 293.8 |  |
| Habitat Type | 309.38 | 3.5 | 0.094 | 3 | 303.38 |  |
| *Fisher: Fall* | | | | | |  |
| Habitat Type +SD | 310.77 | 0 | 0.623 | 7 | 296.77 |  |
| Habitat Type | 311.66 | 0.89 | 0.25 | 3 | 305.66 |  |
| SD | 312.34 | 1.57 | 0.127 | 4 | 304.34 |  |
| *Fisher: Winter* | | | | | |  |
| FHD + SD | 348.23 | 0 | 0.483 | 6 | 336.23 |  |
| Habitat Type | 349.34 | 1.11 | 0.317 | 3 | 343.34 |  |
| FHD +SD + Habitat Type | 349.79 | 1.56 | 0.196 | 9 | 331.79 |  |
| *Raccoon: Spring* | | | | | |  |
| WET | 274.92 | 0 | 0.473 | 4 | 266.92 |  |
| Habitat Type + WET | 276.12 | 1.2 | 0.325 | 7 | 262.12 |  |
| Habitat Type | 277.7 | 2.78 | 0.199 | 3 | 271.7 |  |
| *Raccoon: Summer* | | | | | |  |
| DPR | 268.33 | 0 | 0.731 | 4 | 260.33 |  |
| DPR + Habitat Type | 270.81 | 2.48 | 0.193 | 7 | 256.81 |  |
| Habitat Type | 271.35 | 3.02 | 0.076 | 3 | 265.35 |  |
| *Raccoon: Fall* | | | | | |  |
| Habitat Type | 247.84 | 0 | 0.703 | 3 | 241.84 |  |
| Habitat Type + FOR | 248.77 | 0.93 | 0.265 | 7 | 234.77 |  |
| FOR | 250.24 | 2.4 | 0.031 | 4 | 260.33 |  |
| *Raccoon: Winter* | | | | | |  |
| DPR + WET | 285.75 | 0 | 0.607 | 5 | 275.75 |  |
| DPR +WET + Habitat Type | 287.47 | 1.72 | 0.302 | 8 | 271.47 |  |
| Habitat Type | 288.34 | 2.59 | 0.091 | 3 | 282.34 |  |

^a^ FHD, foliage height diversity; DPR, distance to public road; CC, canopy cover; FOR, percent forest; DSR, distance to skid road; SD, snag density; WET, percent wetland,

^b^ Difference in Akaike’s Information Criterion from the top model to current model

^c^ Model weight (model probability)

^d^ Number of model parameters

^e^ -2Log(Likelihood), measure of model fit

S6. Odds ratios of mesopredator species utilizing each habitat type (shelterwood, field, and reserve forest). The effect habitat type was compared to the reference habitat type in the pairwise comparisons. Estimates are from the top model when *habitat* *type* was included for a species (see S5). We considered significance when the 95% confidence intervals did not overlap with 1. LCL and UCL refer to lower 95% confidence interval and upper 95% confidence interval, respectively.

| Effect Habitat Type | Reference Habitat Type | Odds Ratio | LCL | UCL |  |
| --- | --- | --- | --- | --- | --- |
|  |  |  |  |  |  |
| *Bobcat: Spring* | | | | |  |
| Shelterwood | Reserve | 3.64 | 1.02 | 3.03 |  |
| Field | Reserve | 1.7 | 0.38 | 1.93 |  |
| Shelterwood | Field | 7.04 | 1.36 | 24.90 |  |
| *Bobcat: Winter* | | | | |  |
| Shelterwood | Reserve | 5.803 | 1.15 | 10.55 |  |
| Field | Reserve | 0.36 | 0.07 | 10.62 |  |
| Shelterwood | Field | 4.46 | 1.12 | 4.62 |  |
| *Coyote: Spring* | | | | |  |
| Shelterwood | Reserve | 1.62 | 0.26 | 1.96 |  |
| Field | Reserve | 5.47 | 1.06 | 8.49 |  |
| Field | Shelterwood | 4.28 | 1.04 | 4.18 |  |
| *Fisher: Spring* | | | | |  |
| Reserve | Shelterwood | 1.37 | 0.17 | 2.12 |  |
| Reserve | Field | 4.643 | 1.54 | 5.12 |  |
| Shelterwood | Field | 0.46 | 0.11 | 7.26 |  |
| *Fisher: Fall* | | | | |  |
| Shelterwood | Reserve | 0.55 | 0.06 | 5.59 |  |
| *Reserve* | Field | 3.82 | 1.07 | 3.30 |  |
| Shelterwood | Field | 5.22 | 1.08 | 7.25 |  |
| *Raccoon: Spring* | | | | |  |
| Shelterwood | Reserve | 0.42 | 0.17 | 8.33 |  |
| Field | Reserve | 4.07 | 1.08 | 3.74 |  |
| Field | Shelterwood | 3.95 | 1.05 | 3.52 |  |

S7. Detection probability (*p)* for each species for each season. The null model, with null p(.) and psi(.) is included for comparison. Occupancy was held constant [psi(.)] to estimated *p* for each species, and each season was analyzed separately.

| Model | ΔAIC^a^ | *w*^b^ | K^c^ | -2*LogLik^d^ |  |
| --- | --- | --- | --- | --- | --- |
|  |  |  |  |  |  |
| Coyote: Summer | | | | |  |
|  |  |  |  |  |  |
| *p*(temp) | 0 | 0.483 | 3 | 350.1 |  |
|  |  |  |  |  |  |
| *p*(prevdetect) | 1.07 | 0.196 | 6 | 348.9 |  |
|  |  |  |  |  |  |
| *p(cc)* | 1.89 | 0.125 | 3 | 351.3 |  |
|  |  |  |  |  |  |
| *p*(.) | 2.45 | 0.11 | 2 | 354.14 |  |
|  |  |  |  |  |  |
| *p(precip)* | 8.27 | 0.0067 | 3 | 352.4 |  |
|  |  |  |  |  |  |
| *p(fhd)* | 10.13 | 0.004 | 3 | 347.9 |  |
|  |  |  |  |  |  |
| Coyote: Spring | | | | |  |
|  |  |  |  |  |  |
| *p*(.) | 0 | 0.533 | 2 | 359.58 |  |
|  |  |  |  |  |  |
| *p*(prevdetect) | 0.39 | 0.16 | 6 | 356.23 |  |
|  |  |  |  |  |  |
| *p(temp)* | 1.23 | 0.146 | 3 | 355.81 |  |
|  |  |  |  |  |  |
| *p(cc)* | 1.56 | 0.093 | 3 | 360.11 |  |
|  |  |  |  |  |  |
| *p*(precip) | 2.58 | 0.015 | 3 | 358.95 |  |
|  |  |  |  |  |  |
| *P(fhd)* | 3.92 | 0.002 | 3 | 352.76 |  |
|  |  |  |  |  |  |
| Coyote: Fall | | | | |  |
|  |  |  |  |  |  |
| *p*(precip) | 0 | 0.47 | 3 | 365.33 |  |
|  |  |  |  |  |  |
| *p*(.) | 2.09 | 0.25 | 2 | 362.84 |  |
|  |  |  |  |  |  |
| *p*(temp) | 2.11 | 0.104 | 3 | 361.77 |  |
|  |  |  |  |  |  |
| *p(prevdetect)* | 8.98 | 0.01 | 6 | 370.12 |  |
|  |  |  |  |  |  |
| *p(cc)* | 10.24 | 0.009 | 3 | 367.46 |  |
|  |  |  |  |  |  |
| *P(fhd)* | 11.01 | 0.004 | 3 | 364.98 |  |
|  |  |  |  |  |  |
| Coyote: Winter | | | | |  |
|  |  |  |  |  |  |
| *p*(precip) | 0 | 0.65 | 3 | 372.11 |  |
|  |  |  |  |  |  |
| *p*(temp) | 1.99 | 0.27 | 3 | 374.56 |  |
|  |  |  |  |  |  |
| *p*(.) | 2.76 | 0.02 | 2 | 371.9 |  |
|  |  |  |  |  |  |
| *p(cc)* | 3.25 | 0.014 | 3 | 373.4 |  |
|  |  |  |  |  |  |
| *p(prevdetect)* | 4.51 | 0.008 | 6 | 362.39 |  |
|  |  |  |  |  |  |
| *P(fhd)* | 5.36 | 0.007 | 3 | 375.1 |  |
|  |  |  |  |  |  |
| Bobcat: Spring | | | | |  |
|  |  |  |  |  |  |
| *p*(precip) | 0 | 0.38 | 3 | 142.32 |  |
|  |  |  |  |  |  |
| *p(fhd)* | 0.35 | 0.27 | 3 | 143.45 |  |
|  |  |  |  |  |  |
| *p(prevdetect)* | 0.59 | 0.21 | 6 | 141.22 |  |
|  |  |  |  |  |  |
| *p(.)* | 2.13 | 0.1 | 2 | 140.78 |  |
|  |  |  |  |  |  |
| *p*(temp) | 4.39 | 0.009 | 3 | 145.01 |  |
|  |  |  |  |  |  |
| *p(cc)* | 6.28 | 0.008 | 3 | 148.56 |  |
|  |  |  |  |  |  |
| Bobcat: Summer | | | | |  |
|  |  |  |  |  |  |
| *p(fhd)* | 0 | 0.31 | 3 | 154.81 |  |
|  |  |  |  |  |  |
| *p(.)* | 0.37 | 0.23 | 2 | 155.5 |  |
|  |  |  |  |  |  |
| *p(temp)* | 0.8 | 0.14 | 3 | 156.75 |  |
|  |  |  |  |  |  |
| *p*(prevdetect) | 2.12 | 0.106 | 6 | 152.93 |  |
|  |  |  |  |  |  |
| *p*(cc) | 3.6 | 0.09 | 3 | 151.18 |  |
|  |  |  |  |  |  |
| *p(temp)* | 3.7 | 0.045 | 3 | 153.4 |  |
|  |  |  |  |  |  |
| Bobcat: Fall | | | | |  |
|  |  |  |  |  |  |
| *p(temp)* | 0 | 0.364 | 3 | 139.24 |  |
|  |  |  |  |  |  |
| *p(precip)* | 0.57 | 0.22 | 3 | 138.13 |  |
|  |  |  |  |  |  |
| *p*(fhd) | 0.78 | 0.19 | 3 | 133.45 |  |
|  |  |  |  |  |  |
| *p(prevdetect)* | 1.02 | 0.11 | 6 | 134.21 |  |
|  |  |  |  |  |  |
| *p*(cc) | 2.11 | 0.09 | 6 | 135.23 |  |
|  |  |  |  |  |  |
| *p*(.) | 3.25 | 0.009 | 2 | 140.79 |  |
|  |  |  |  |  |  |
| Bobcat: Winter | | | | |  |
|  |  |  |  |  |  |
| *p(precip)* | 0 | 0.423 | 3 | 180.3 |  |
|  |  |  |  |  |  |
| *p(temp)* | 1.9 | 0.201 | 3 | 176.07 |  |
|  |  |  |  |  |  |
| *p*(.) | 2.07 | 0.17 | 2 | 173.43 |  |
|  |  |  |  |  |  |
| *p(cc)* | 2.85 | 0.11 | 3 | 181.56 |  |
|  |  |  |  |  |  |
| *p(fhd)* | 3.66 | 0.08 | 3 | 174.39 |  |
|  |  |  |  |  |  |
| *p*(prevdetect) | 6.05 | 0.013 | 6 | 177.42 |  |
|  |  |  |  |  |  |
| Raccoon: Spring | | | | |  |
|  |  |  |  |  |  |
| *p*(temp) | 0 | 0.25 | 3 | 330.49 |  |
|  |  |  |  |  |  |
| *p*(.) | 0.22 | 0.212 | 2 | 333.05 |  |
|  |  |  |  |  |  |
| *p(cc)* | 0.9 | 0.177 | 3 | 326.73 |  |
|  |  |  |  |  |  |
| *p(precip)* | 1.43 | 0.135 | 3 | 326.91 |  |
|  |  |  |  |  |  |
| *p(prevdetect)* | 2.65 | 0.101 | 6 | 332.1 |  |
|  |  |  |  |  |  |
| *p(fhd)* | 3.9 | 0.092 | 3 | 329.34 |  |
|  |  |  |  |  |  |
| Raccoon: Summer | | | | |  |
|  |  |  |  |  |  |
| *p*(cc) | 0 | 0.482 | 3 | 383.46 |  |
|  |  |  |  |  |  |
| *p*(precip) | 0.14 | 0.223 | 3 | 385.11 |  |
|  |  |  |  |  |  |
| *p*(temp) | 1.23 | 0.13 | 3 | 381.32 |  |
|  |  |  |  |  |  |
| *p(.)* | 2.1 | 0.101 | 2 | 382.44 |  |
|  |  |  |  |  |  |
| *p(prevdetect)* | 2.89 | 0.01 | 6 | 380.25 |  |
|  |  |  |  |  |  |
| *p(fhd)* | 4.29 | 0.007 | 3 | 381.22 |  |
|  |  |  |  |  |  |
| Raccoon: Fall | | | | |  |
|  |  |  |  |  |  |
| *p*(temp) | 0 | 0.394 | 3 | 365.11 |  |
|  |  |  |  |  |  |
| *p*(.) | 0.25 | 0.28 | 2 | 363.29 |  |
|  |  |  |  |  |  |
| *p(cc)* | 1.3 | 0.18 | 3 | 367.98 |  |
|  |  |  |  |  |  |
| *p(precip)* | 1.91 | 0.07 | 3 | 362.76 |  |
|  |  |  |  |  |  |
| *p(fhd)* | 2.24 | 0.064 | 3 | 364.21 |  |
|  |  |  |  |  |  |
| *p(prevdetect)* | 2.95 | 0.003 | 6 | 362.54 |  |
|  |  |  |  |  |  |
| Raccoon: Winter | | | | |  |
|  |  |  |  |  |  |
| *p*(temp) | 0 | 0.49 | 3 | 320.87 |  |
|  |  |  |  |  |  |
| *p(precip)* | 1.41 | 0.161 | 3 | 318.42 |  |
|  |  |  |  |  |  |
| *p*(.) | 1.76 | 0.15 | 2 | 323.76 |  |
|  |  |  |  |  |  |
| *p(cc)* | 1.99 | 0.1 | 6 | 314.85 |  |
|  |  |  |  |  |  |
| *p(prevdetect)* | 3.6 | 0.09 | 6 | 321.23 |  |
|  |  |  |  |  |  |
| *p(fhd)* | 4.5 | 0.005 | 3 | 318.1 |  |
|  |  |  |  |  |  |
| Fisher: Spring | | | | |  |
|  |  |  |  |  |  |
| *p*(*prevdetect*) | 0 | 0.423 | 6 | 125.93 |  |
|  |  |  |  |  |  |
| *p*(cc) | 0.21 | 0.24 | 3 | 124.89 |  |
|  |  |  |  |  |  |
| *p*(precip) | 0.24 | 0.18 | 3 | 120.6 |  |
|  |  |  |  |  |  |
| *p(.)* | 0.98 | 0.11 | 2 | 132.31 |  |
|  |  |  |  |  |  |
| *p(fhd)* | 1.13 | 0.011 | 3 | 127.43 |  |
|  |  |  |  |  |  |
| *p(temp)* | 1.8 | 0.003 | 3 | 128.31 |  |
|  |  |  |  |  |  |
| Fisher: Summer | | | | |  |
|  |  |  |  |  |  |
| *p*(cc) | 0 | 0.35 | 3 | 130.43 |  |
|  |  |  |  |  |  |
| *p*(.) | 0.15 | 0.21 | 2 | 126.52 |  |
|  |  |  |  |  |  |
| *p*(precip) | 0.21 | 0.19 | 3 | 127.34 |  |
|  |  |  |  |  |  |
| *p(prevdetect)* | 0.87 | 0.12 | 6 | 134.81 |  |
|  |  |  |  |  |  |
| *p(temp)* | 1.02 | 0.11 | 3 | 129.92 |  |
|  |  |  |  |  |  |
| *p(fhd)* | 2.4 | 0.009 | 3 | 134.5 |  |
|  |  |  |  |  |  |
| Fisher: Fall | | | | |  |
|  |  |  |  |  |  |
| *p*(precip) | 0 | 0.443 | 3 | 150.28 |  |
|  |  |  |  |  |  |
| *p*(cc) | 0.74 | 0.23 | 3 | 151.23 |  |
|  |  |  |  |  |  |
| *p*(prevdetect) | 0.92 | 0.17 | 6 | 152.76 |  |
|  |  |  |  |  |  |
| *p(temp)* | 1.56 | 0.12 | 3 | 153.38 |  |
|  |  |  |  |  |  |
| *p(fhd)* | 1.89 | 0.0149 | 3 | 148.57 |  |
|  |  |  |  |  |  |
| *p(.)* | 2.01 | 0.005 | 2 | 152.26 |  |
|  |  |  |  |  |  |
| Fisher: Winter | | | | |  |
|  |  |  |  |  |  |
| *p*(prevdetect) | 0 | 0.462 | 6 | 123.71 |  |
|  |  |  |  |  |  |
| *p*(fhd) | 0.87 | 0.25 | 3 | 124.23 |  |
|  |  |  |  |  |  |
| *p(precip)* | 1.15 | 0.23 | 3 | 127.25 |  |
|  |  |  |  |  |  |
| *p(temp)* | 1.33 | 0.02 | 3 | 120.64 |  |
|  |  |  |  |  |  |
| *p(.)* | 2.12 | 0.01 | 2 | 129.09 |  |
|  |  |  |  |  |  |
| *P(cc)* | 2.5 | 0.008 | 3 | 124.9 |  |
|  |  |  |  |  |  |

Difference in Akaike’s Information Criterion from the top model to current model

Model weight (model probability)

Number of model parameters

-2Log(Likelihood), measure of model fit
